# Electrophysiological evidence of subclinical trigeminal dysfunction in patients with COVID-19 and smell impairment: A pilot study

**DOI:** 10.3389/fneur.2022.981888

**Published:** 2022-10-14

**Authors:** Giuseppe Cosentino, Eugenia Maiorano, Massimiliano Todisco, Paolo Prunetti, Elisa Antoniazzi, Giulia Tammam, Ilaria Quartesan, Sara Lettieri, Roberto De Icco, Angelo Guido Corsico, Marco Benazzo, Antonio Pisani, Cristina Tassorelli, Enrico Alfonsi

**Affiliations:** ^1^Department of Brain and Behavioral Sciences, University of Pavia, Pavia, Italy; ^2^Translational Neurophysiology Research Unit, IRCCS Mondino Foundation, Pavia, Italy; ^3^Department of Otolaryngology–Head and Neck Surgery, Fondazione IRCCS Policlinico San Matteo, Pavia, Italy; ^4^Department of Internal Medicine and Therapeutics, University of Pavia, Pavia, Italy; ^5^Headache Science and Neurorehabilitation Center, IRCCS Mondino Foundation, Pavia, Italy; ^6^Pneumology Unit, IRCCS San Matteo Hospital Foundation, Pavia, Italy; ^7^Department of Clinical, Surgical, Diagnostic and Pediatric Sciences, University of Pavia, Pavia, Italy; ^8^Movement Disorders Research Center, IRCCS Mondino Foundation, Pavia, Italy

**Keywords:** COVID-19, SARS-CoV-2, trigeminal nerve, anosmia, brainstem reflexes

## Abstract

**Background:**

Smell and taste disturbances are among the most frequent neurological symptoms in patients with COVID-19. A concomitant impairment of the trigeminal nerve has been suggested in subjects with olfactory dysfunction, although it has not been confirmed with objective measurement techniques. In this study, we explored the trigeminal function and its correlations with clinical features in COVID-19 patients with impaired smell perception using electrophysiological testing.

**Methods:**

We enrolled 16 consecutive patients with mild COVID-19 and smell impairment and 14 healthy controls (HCs). Olfactory and gustatory symptoms were assessed with self-reported questionnaires. Electrophysiological evaluation of the masseter inhibitory reflex (MIR) and blink reflex (BR) was carried out to test the trigeminal function and its connections within the brainstem.

**Results:**

Masseter inhibitory reflex (MIR) analysis revealed higher latency of ipsilateral and contralateral early silent period in patients when compared with HCs. No significant differences between groups were detected as regards the duration of the early and late silent period. However, several patients showed a prolonged duration of the early silent period. BR evaluation disclosed only an increased amplitude of early components in patients.

**Conclusions:**

Patients with COVID-19 and smell impairment show a subclinical trigeminal nerve impairment. Trigeminal alterations mainly involve the oligosynaptic pathway, as a result of either direct viral damage or secondary neuroinflammation of the peripheral trigeminal fibers, whereas the polysynaptic ponto-medullary circuits seem to be spared. The prolonged duration of the early silent period and the increased amplitude of early BR response might reflect a compensatory upregulation of the trigeminal function as a consequence of the olfactory dysfunction.

## Introduction

Neurological symptoms are common in patients with COVID-19, with an incidence of up to 80% in hospitalized patients (1, 2). Neurological impairment has also been identified as a risk factor for mortality in this patient population (2, 3). Headache, anosmia, and ageusia are the most frequent neurological symptoms, whereas acute encephalopathy, coma, and stroke represent the most common neurological syndromes (2, 4). Beyond the loss of smell and taste sensation, many other symptoms due to cranial nerve involvement have been reported in patients with COVID-19, including sudden vision and hearing loss, peripheral vertigo, swallowing disorders, hoarseness, eye movement limitation, and facial hypoesthesia (5–7). It remains unclear whether these symptoms are a consequence of the direct viral invasion of the nervous system or represent an epiphenomenon of the immune system response triggered by the severe acute respiratory syndrome coronavirus 2 (SARS-CoV-2) (8–10).

Evidence has been provided that cranial nerve sensory functions might be particularly affected in patients with COVID-19 (11). In this context, a trigeminal impairment associated with SARS-CoV-2 infection may not be surprising, in consideration of clinical studies showing that, along with the olfactory dysfunction, the chemesthetic sensations mediated by the trigeminal fibers can also be affected during COVID-19 (12, 13). However, due to the subjective nature of the clinical testing, findings of trigeminal impairment remain to be confirmed by objective measurement methods.

In this study, we aimed at assessing the trigeminal function in a cohort of patients with COVID-19 using an electrodiagnostic approach. We focused on subjects with hyposmia or anosmia based on the suggestion of a more marked neurotropism of SARS-CoV-2 in this patient's subgroup (12, 14, 15). Electrophysiological evaluation of the masseter inhibitory reflex (MIR) and blink reflex (BR) was carried out to test the functional integrity of the trigeminal nerve fibers as well as the excitatory and inhibitory inter-neuronal brainstem pathways. Moreover, the electrophysiological findings were related to clinical data and a subgroup of patients with COVID-19 was followed up to longitudinally evaluate the prognostic significance of the electrophysiological changes.

## Methods

### Study population

The present study was conducted at the IRCCS Mondino Foundation (Pavia, Italy) and the Otolaryngology Department of the IRCCS Policlinico San Matteo Foundation (Pavia, Italy). Sixteen patients with COVID-19 and decrease in smell sensation (hyposmia or anosmia) (mean age ± SD: 44.0 ± 12.0 years, six men) and 14 age-matched healthy subjects (mean age ± SD: 41.3 ± 9.5 years, seven men) were enrolled. Patients were consecutively recruited from an otolaryngology outpatient clinic dedicated to olfactory disorders, between March and May 2020 during the first COVID-19 outbreak wave. Inclusion criteria were: SARS-CoV-2 detection by polymerase chain reaction (PCR) through a nasopharyngeal swab at first assessment, age of 18 years or above, and a new-onset olfactory disorder. Exclusion criteria were: pre-existing olfactory or gustatory disorders and nasal diseases (e.g., previous local trauma, chronic sinusitis, or acute allergic symptoms), substance abuse (including nasal decongestant drugs), neuropsychiatric disorders, major head and neck traumatic injuries, and previous chemotherapy or radiation therapy of the head and neck region. At the time of enrollment, all participants underwent a baseline interview assessing general demographic and clinical variables (Table 1). A thorough neurological and ear-nose-throat (ENT) physical examination was conducted for all participants. Endoscopic evaluation was not performed to prevent the potential aerosolization of viral particles.

**Table 1:** Demographic and clinical characteristics of subjects enrolled.

	**All patients (*n* = 16)**	**Patients who underwent T2 (*n* = 7)**	**Healthy subjects (*n* = 14)**
Sex	6M/10F	3M/4F	7M/7F
Age, years	44.0 ± 12.4	43.6 ± 10.7	41.1 ± 9.8
Interval onset-EDX, days	16.0 ± 12.0	12.9 ± 5.5	-
VAS-O (T0)	8.7 ± 2.4	8.4 ± 2.8	-
HRS (T0)	26.2 ± 6.6	25.7 ± 7.7	-
VAS-G (T0)	7.3 ± 4.0	6.7 ± 4.7	-
CiTAS (T0)	28.8 ± 12.3	28.4 ± 14.2	-
VAS-O (T1)	2.4 ± 3.7	2.4 ± 4.2	-
HRS (T1)	13.2 ± 9.5	12.0 ± 10.4	-
VAS-G (T1)	2.7 ± 3.9	2.4 ± 4.2	-
CiTAS (T1)	15.5 ± 11.4	14.4 ± 12.3	-
VAS-O (T2)	-	0.6 ± 1.5	-
HRS (T2)	-	7.7 ± 4.5	-
VAS-G (T2)	-	0.6 ± 1.5	-
CiTAS (T2)	-	10.3 ± 6.0	-

Data are reported as number of patients or mean values ± SD.

CiTAS, Chemotherapy Induced Taste Alteration Scale; EDX, electrodiagnostic examination; HRS, Hyposmia Rating Scale; T0, baseline assessment; T1, 1-month follow-up; T2, 20-month follow-up; VAS-G, Gustatory Visual Analog Scale; VAS-O, Olfactory Visual Analog Scale.

### Clinical assessment of the olfactory and gustatory function

Since psychophysical tests were not practicable during the COVID-19 emergency state, only subjective olfactory and gustatory symptoms were assessed. Clinical follow-up was performed after 1 month in all patients and after 20 months in a subgroup of seven patients. A 10-cm Visual Analog Scale (VAS), anchored at each end with verbal descriptors (“no impairment−0” and “extreme impairment−10”), was administered to investigate both olfactory (VAS-O) and gustatory (VAS-G) dysfunctions. VAS has been already employed for the assessment of olfactory and gustatory functions (16), and significant correlations have been found between this scale and the Objective Odor Stick Tests (OOST) (17, 18).

Two additional patient-reported questionnaires were also administered: the Hyposmia Rating Scale (HRS) and the Chemotherapy Induced Taste Alteration Scale (CiTAS) (19). The HRS has been originally developed to grade olfactory dysfunctions in Parkinson's disease (20), and its clinical utility has been tested in several settings, showing a strong correlation with the OOST (21, 22). The total HRS score ranges from 6 (“no impairment”) to 30 (“worst impairment”). Taste alterations were assessed using the Italian validated version of the CiTAS, an 18-item scale that explores specific taste disturbances and their impact on patient nutrition (23, 24). The total CiTAS score ranges from 8 (“no impairment”) to 40 (“worst impairment”).

### Electrophysiological assessment

Masseter inhibitory reflex (MIR) and BR were assessed in a single experimental session using a 4-channel electromyography device and dedicated software (Medelec Synergy, CareFusion Corporation, San Diego, CA). All patients and controls completed the planned electrophysiological investigations at baseline. A subgroup of seven patients underwent a follow-up evaluation 20 months after the baseline. The assessments were performed in a noiseless laboratory at normal room temperature, with patients lying on the examination table in a supine position with their eyes closed. Surface electrodes were used for electrophysiological testing. MIR and BR were recorded according to the Recommendations of the International Federation of Clinical Neurophysiology (25).

For the MIR investigation, the activity of both masseter muscles was tracked in two channels with the active electrode placed over the muscle belly and the reference electrode on the cheekbone arch. The mental nerve was stimulated by means of electrical stimuli with a duration of 0.2 ms in the projection of the mental foramen during maximal voluntary contraction of masticatory muscles. To avoid reflex habituation, stimulation was performed at random intervals ranging from 60 to 120 s. The stimulus intensity was eight times the sensory threshold (range from 8 to 23 mA), and it was usually felt as mildly painful (3–4 on a 10-degree VAS). The right and the left nerve were stimulated separately and consecutively. Eight traces with a duration of 250 ms and a pre-stimulus delay of 50 ms were averaged. Latencies at the onset and duration of the early (SP1) and late silent period (SP2) were assessed. The onset of the CSP was defined as the time when the electromyography (EMG) fell below 50% of the background EMG level preceding stimulus for at least 10 ms, and the end of the silent period was defined as when EMG returned to 50% of baseline for at least 10 ms (26, 27).

For BR examination, the active electrodes were placed bilaterally on the orbicularis oculi muscle and the reference electrodes just laterally to the lateral canthus. Right and left supraorbital nerves were stimulated successively over the supraorbital foramen. Electrical stimuli with a duration of 0.2 ms were applied at random intervals ranging from 60 to 120 s. Responses were elicited eight times, consecutively from both sides, and we recorded the following components: early component (R1), ipsilateral (iR2), and contralateral late component (cR2). The latency of reflex responses was measured from the shortest initial deflection, the amplitude was calculated from onset to maximum peak, and the duration was determined by manual cursor marking from the beginning to the end of responses. Average values of latency, duration, and amplitude of R1, iR2, and cR2 were calculated.

Signals were stored for offline analysis (band-pass filter: 10 Hz−10 kHz; sensitivity: 200 μV/Div; sweep speed: 10 ms/Div). Two of the authors, blind to the subjects' group, performed the electrophysiological evaluations and calculated MIR and BR measures.

### Statistical analyses

The Kolmogorov-Smirnov normality test was applied for all electrophysiological variables. Mann–Whitney U test was used if the test was significant; otherwise, Student's *t*-test was carried out. Pearson's test was used to test correlations between clinical and electrophysiological data. In order to avoid a type II error in this exploratory study, we did not use correction for multiple comparisons.

For all analyses, *p* < 0.05 was considered significant. Statistical analyses were performed using SPSS statistical software (version 21.0).

## Results

All but two patients had an overall mild disease course that did not require hospitalization. In most cases, the decrease in smell sensation (anosmia in 14 patients, hyposmia in two patients) represented the main symptom for which the patients were referred to the ENT outpatient clinic. Ageusia and hypogeusia were present in 11 and four patients, respectively. The onset of smell and taste disturbances was acute in all patients and cacosmia was not reported in any patient.

Additional symptoms included fever (ten patients), asthenia (ten patients), cough (two patients), nasal obstruction and/or rhinorrhea (six patients), dyspnea (two patients), musculoskeletal pain (two patients), and diarrhea (two patients). In addition to smell and taste disturbances, 11 patients reported other neurological symptoms, which included headache (nine patients) and limb paraesthesia (two patients). Neurological examination was, however, normal in all patients. None of the patients had been admitted to the intensive care unit. Therapy made included hydroxychloroquine (three patients), paracetamol (five patients), antibiotic therapy (three patients), and NSAIDs (one patient); no patient had practiced topical or systemic steroid therapy. None of the patients had previously been vaccinated as the experiment was conducted before the anti-COVID-19 vaccination campaign began. No patient had a undergone brain MRI at the time of the experiment. The time interval between the onset of disease symptoms and the electrophysiological assessment ranged from 5 to 50 days (mean ± SD: 16.0 ± 11.9 days). Clinical improvement was observed in all but two patients at 1-month follow-up (*p* < 0.01 for all clinical scales) (Table 1). Mean values together with the 95% confidence interval of the electrophysiological measures recorded in patients and healthy controls (HCs) are reported in Tables 2, 3. We refer to the 95% confidence interval found in the healthy subjects' group as the normative value range. Individual electrophysiological data compared with normal values are reported in Tables 4, 5.

**Table 2:** Findings from the masseter inhibitory reflex in COVID-19 patients and healthy subjects.

			**Patients**	**Healthy controls**
**Latency, ms**	Right stimulation	iSP1	15.1 ± 3.2; 11.7–21.6^*^	12.4 ± 1.9; 9.3–17.1
		cSP1	15.8 ± 3.5; 10.5–21.9^**^	11.4 ± 0.8; 10.5–13.2
		iSP2	49.8 ± 7.9; 37.2–64.5	48.8 ± 8.1; 33.6–60.6
		cSP2	50.2 ± 8.4; 38.4–65.7	48.5 ± 7.8; 33.6–57.3
	Left stimulation	iSP1	15.4 ± 3.6; 11.7–24.9^**^	11.8 ± 1.2; 9.6–14.4
		cSP1	15.1 ± 3.2; 10.5–23.1^**^	12.0 ± 1.4; 10.2–15.3
		iSP2	48.0 ± 8.4; 34.5–62.1	49.4 ± 6.2; 40.8–61.5
		cSP2	48.5 ± 7.8; 32.7–61.8	48.4 ± 7.0; 37.8–63.0
**Duration, ms**	Right stimulation	iSP1	18.5 ± 11.0; 9.6–56.1	14.5 ± 2.7; 10.2–18.9
		cSP1	17.8 ± 11.4; 9.6–56.1	13.6 ± 3.6; 9.0–23.3
		iSP2	34.0 ± 8.7; 22.2–52.8	35.7 ± 8.3; 24–50.1
		cSP2	33.1 ± 10.1; 19.2–53.4	38.5 ± 8.4; 25.2–48.6
	Left stimulation	iSP1	19.0 ± 12.2; 10.5–59.4	14.3 ± 2.7; 10.2–18.6
		cSP1	19.7 ± 11.2; 12.0–54.6	14.9 ± 2.9; 10.2–19.2
		iSP2	34.3 ± 8.8; 25.5–57.0	39.7 ± 8.3; 19.5–49.8
		cSP2	35.6 ± 8.2; 21.9–54.3	39.4 ± 4.6; 33.3–48.9

Mean values ± SD and 95% confidence intervals are showed. The 95% confidence intervals recorded in the healthy controls correspond to the normative value range.

* p < 0.05,

** p < 0.01.

cSP1, contralateral early silent period; cSP2, contralateral late silent period; iSP1, ipsilateral early silent period; iSP2, ipsilateral late silent period.

**Table 3:** Findings from the blink reflex in COVID-19 patients and healthy subjects.

			**Patients**	**Healthy controls**
**Latency, ms**	Right stimulation	R1	11.1 ± 1.0; 9.5–13.1	11.0 ± 1.1; 7.9–12.1
		iR2	32.2 ± 3.2; 25.9–36.5	32.2 ± 3.9; 23.3–38.3
		cR2	33.5 ± 3.0; 28.3–37.6	33.3 ± 4.2; 24.5–41.6
	Left stimulation	R1	11.1 ± 1.2; 9.2–13.2	10.8 ± 1.1; 8.9–12.1
		iR2	32.5 ± 3.8; 26.4–40.7	32.2 ± 4.0; 22.5–37.0
		cR2	32.7 ± 4.2; 25.1–41.0	34.1 ± 4.4; 23.7–40.4
**Amplitude**, **μV**	Right stimulation	R1	227.5 ± 128.3; 96.4–458.1^*^	115.0 ± 100.2; 21.4–336.0
		iR2	224.3 ± 89.1; 109.7–378.6	168.3 ± 86.7; 5.4–303.6
		cR2	168.3 ± 86.3; 45.2–406.5	154.9 ± 107.3; 35.7–396.4
	Left stimulation	R1	193.1 ± 108.1; 35.5–419.4	134.3 ± 84.9; 17.9–307.1
		iR2	205.5 ± 92.9; 72.9–361.3	213.6 ± 98.2; 66.1–357.1
		cR2	189.2 ± 79.1; 72.6–354.8	150.3 ± 58.6; 51.8–221.4
**Duration, ms**	Right stimulation	R1	8.1 ± 1.4; 6.3–12.0	7.7 ± 1.4; 5.1–9.9
		iR2	44.1 ± 6.4; 36.0–59.3^**^	37.3 ± 4.6; 30.3–45.4
		cR2	42.3 ± 6.7; 33.1–57.5^*^	36.3 ± 6.6; 26.0–48.5
	Left stimulation	R1	8.0 ± 1.1; 6.3–10.2	8.0 ± 1.8; 5.2–12.1
		iR2	41.7 ± 4.3; 35.8–48.5	41.9 ± 3.9; 36.4–51.1
		cR2	41.8 ± 3.7; 34.4–47.1	40.0 ± 5.0; 1.5–49.0

Mean values ± SD and 95% confidence intervals are showed. The 95% confidence intervals recorded in the healthy controls correspond to the normative value range.

* p < 0.05,

** p < 0.01.

iR2, ipsilateral R2; cR2, contralateral R2.

**Table 4:** Individual parameters of the masseter inhibitory reflex in COVID-19 patients as compared to healthy subjects at baseline and at 20-month follow-up.

	**T0**		**T2**	
**Patients**	**Latency**	**Duration**	**Latency**	**Duration**
1	↑ cSP1 after right stimulation; ↑ iSP1 and cSP1 after left stimulation	↑ iSP1 and cSP1 after bilateral stimulation	-	-
2	↑ iSP1 and cSP1 after left stimulation; ↑ iSP2 and cSP2 after right stimulation	↑ iSP1 after right stimulation; ↓ iSP2 and cSP2 after right stimulation	-	-
3	↑ iSP1 after right stimulation; absent iSP1 and cSP1 after left stimulation	↓ iSP1 after right stimulation; absent iSP1 and cSP1 after left stimulation	N for iSP1 and cSP1 after right stimulation; ↑ iSP1 after left stimulation	N for iSP1 and cSP1 after bilateral stimulation
4	↑ iSP1 and cSP1 after bilateral stimulation	↑ iSP2 and cSP2 after right stimulation; ↑ cSP2 after left stimulation	-	-
5	↑ iSP1 and cSP1 after left stimulation	↓ cSP2 after left stimulation	-	-
6	↑ iSP1 and cSP1 after bilateral stimulation	↓ cSP2 after left stimulation	-	-
7	↑ cSP1 after right stimulation	N	↑ iSP1 and cSP2 after right stimulation; ↑ cSP1 after left stimulation	↓ cSP2 after left stimulation
8	↑ cSP1 after right stimulation	↓ cSP2 after left stimulation	↑ iSP1 after left stimulation	↓ cSP2 after bilateral stimulation
9	↑ cSP1 after right stimulation	↑ iSP1 and cSP1 after left stimulation; ↓ cSP2 after left stimulation	N	N
10	↑ iSP1 after right stimulation; ↑ iSP1 and cSP1 after left stimulation	N	↑ cSP1 after right stimulation; N for iSP1 and cSP1 after left stimulation	↑ iSP1 after right stimulation; ↑ cSP1 after bilateral stimulation
11	↑ cSP1 after right stimulation; ↑ iSP1 and iSP2 after left stimulation	↑ iSP1 and cSP1 after left stimulation	-	-
12	↑ cSP1 after right stimulation	↑ iSP2 and cSP2 after left stimulation	-	-
13	↑ cSP1 and cSP2 after right stimulation	↑ iSP1 and cSP1 after left stimulation	N for iSP1 and cSP1 after right stimulation; ↑ cSP2 after right stimulation	↑ iSP1 after bilateral stimulation; N for cSP1 after bilateral stimulation
14	Absent SP1 and SP2 after bilateral stimulation	Absent SP1 and SP2 after bilateral stimulation	N	N
15	↑ cSP1 after right stimulation	↑ iSP1 after bilateral stimulation	-	-
16	N	↑ iSP1 after bilateral stimulation; ↑ cSP2 after left stimulation	-	-

Parameters were considered abnormal when their value fell outside the 95% confidence interval recorded in the healthy controls.

cSP1, contralateral early silent period; cSP2, contralateral late silent period; iSP1, ipsilateral early silent period; iSP2, ipsilateral late silent period; N, normal values; T0, baseline assessment; T2, 20-month follow-up; ↑, increased values; ↓, reduced values.

**Table 5:** Individual parameters of the blink reflex in COVID-19 patients as compared to healthy subjects at baseline and at 20-month follow-up.

	**T0**			**T2**		
**Patients**	**Latency**	**Amplitude**	**Duration**	**Latency**	**Amplitude**	**Duration**
1	N	N	↑ iR2 and cR2 after right stimulation	-	-	-
2	N	N	↓ iR2 after left stimulation	-	-	-
3	N	↑ iR2 after right stimulation	↑ iR2 and cR2 after right stimulation	N	N	↑ iR2 and cR2 after right stimulation
4	N	N	↑ iR2 after right stimulation	-	-	-
5	N	↑ iR2 after right stimulation; ↑ R1 and cR2 after left stimulation	N	-	-	-
6	N	N	N	-	-	-
7	↑ R1, iR2, and cR2 after left stimulation	N	N	N	N	N
8	N	↑ R1 after bilateral stimulation	N	N	N	N
9	↑ R1 after left stimulation	↑ R1 after right stimulation	N	N	N	N
10	N	↑ iR2 and cR2 after left stimulation	N	N	N	N
11	N	↑ R1 after right stimulation	N	-	-	-
12	N	N	↑ iR2 after right stimulation	-	-	-
13	N	↑ cR2 after left stimulation	N	N	N	N
14	N	N	N	N	N	N
15	N	↑ iR2 after right stimulation; ↑ iR2 and cR2 after left stimulation	↑ iR2 after right stimulation	-	-	-
16	↑ R1 after right stimulation; ↑ iR2 after left stimulation	N	N	-	-	-

Parameters were considered abnormal when their value fell outside the 95% confidence interval recorded in the healthy controls.

iR2, ipsilateral R2; cR2, contralateral R2; N, normal values; T0, baseline assessment; T2, 20-month follow-up; ↑, increased values.

### Masseter inhibitory reflex

**Figure 1:**
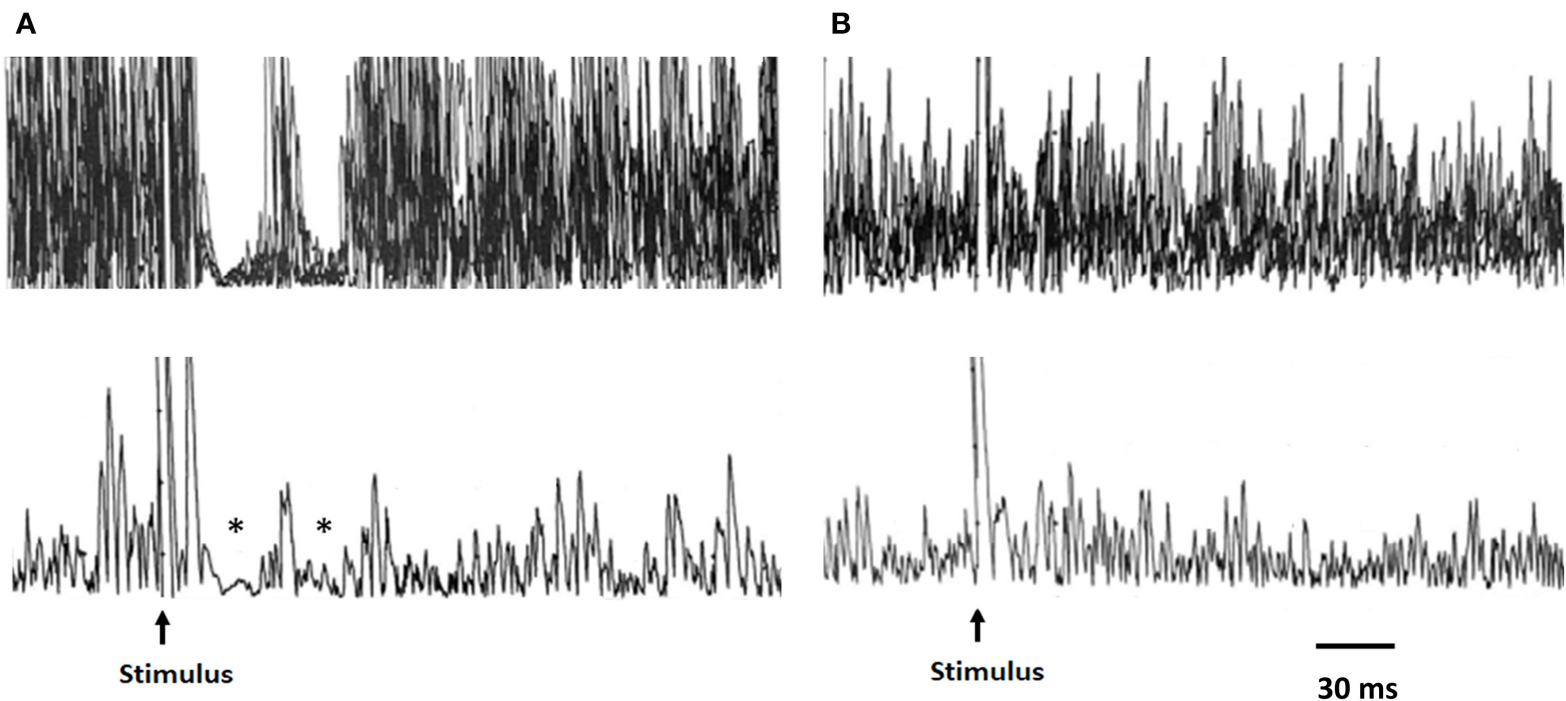
Representative traces of the MIR recordings from a healthy subject and a patient with COVID-19. Upper traces: raw EMG signal, superimposed traces; lower traces: rectified average of same traces. In the healthy subject **(A)**, the stimulation of the mental nerve evokes two silent periods (SP1 and SP2) in the active masseter muscle as indicated by the asterisks. **(B)** Absence of the inhibitory response (silent period) after mental nerve stimulation in a patient evaluated at baseline. MIR, masseter inhibitory reflex.

Patients and HCs did not differ in terms of background electromyographic activity of masseter muscles during voluntary contraction prior to the electrical stimulation (Figures 1A,B). Conversely, as compared with controls, patients showed an increased latency of the ipsilateral (iSP1) and contralateral SP1 (cSP1) after both right (*p* < 0.05 and *p* < 0.01 for the iSP1 and cSP1, respectively) and left stimulation (*p* < 0.01 for both the iSP1 and cSP1) (Table 2). No significant differences were found between the two groups regarding other measures evaluated. Of note, for stimulation on the right side, the SP2 was bilaterally absent in five patients and five healthy subjects, and contralaterally absent in one patient. When stimulating the left side, the SP2 was bilaterally absent in three patients and three healthy subjects, and ipsilaterally absent in one patient.

Single-subject analyses of data recorded at baseline showed at least one measure outside the normative value range in all patients (Table 4). We observed that in one patient, both the SP1 and SP2 were absent after bilateral stimulation, while in another patient, both the SP1 and SP2 were absent only after unilateral stimulation (Figure 1B). In all but one patient, there was an increased latency of the iSP1 or cSP1 after unilateral or bilateral stimulation. Duration of the iSP1 or cSP1 was prolonged after unilateral or bilateral stimulation in seven patients.

### Blink reflex

No differences were observed between patients and controls regarding latency of all responses (i.e., R1, iR2, and cR2) after bilateral stimulation. Instead, as compared with healthy subjects, the patients' cohort had a greater amplitude of the R1 (*p* < 0.05) and an increased duration of the iR2 (*p* < 0.01) and cR2 (*p* < 0.05) after right stimulation (Table 3).

Single-subject analyses of data recorded at baseline showed that at least one electrophysiological measure was outside the normative value range in 14 patients (Table 5). Indeed, an increased latency of the R1 was observed after unilateral stimulation in three patients. The increased amplitude of the R1, iR2, or cR2 was found in eight patients. A prolonged duration of the iR2 or cR2 was observed after unilateral stimulation in five patients.

### Correlation analyses

Significant correlations between clinical and electrophysiological findings are shown in Table 6. Of note, we found a positive correlation between the duration of both the iSP1 and cSP1 at baseline MIR investigation after bilateral stimulation and the time interval between symptom onset and electrodiagnostic examination. We also showed positive correlations between the duration of the iSP1 or cSP1 and scores at clinical scales performed at 1-month follow-up (Table 6) but not at baseline. BR measures did not show any significant correlation.

**Table 6:** Findings from correlation analyses between clinical and electrophysiological findings.

	**d-iSP1 (R)**	**d-cSP1 (R)**	**d-iSP1 (L)**	**d-cSP1 (L)**	**d-cSP2 (R)**	**l-iSP2 (R)**	**l-cSP2 (R)**	**l-iSP2 (L)**	**l-cSP2 (L)**
Interval onset-EDX	*r* = 0.670 *p* = 0.006	*r* =0.753 *p* = 0.002	*r* = 0.649 *p* = 0.012	*r* = 0.565 *p* = 0.035	-	-	-	-	-
VAS-O (T0)	-	-	-	-	*r* = 0.787 *p* = 0.007	-	-	-	-
VAS-G (T0)	-	-	-	-	*r* = 0.649 *p* = 0.042	-	-	-	-
HRS (T0)	-	-	-	-	*r* = 0.726 *p* = 0.017	-	-	-	-
CiTAS (T0)	-	-	-	-	-	-	-	-	-
VAS-O (T1)	*r* = 0.549 *p* = 0.034	-	*r* = 0.703 *p* = 0.005	*r* = 0.767 *p* = 0.001	-	-	-	-	-
VAS-G (T1)	*r* = 0.517 *p* = 0.048	-	*r* = 0.668 *p* = 0.009	*r* = 0.755 *p* = 0.002	-	-	-	-	-
HRS (T1)	-	-	*r* = 0.619 *p* = 0.018	*r* = 0.680 *p* = 0.007	-	-	-	-	-
CiTAS (T1)	*r* = 0.596 *p* = 0.019	*r* = 0.560 *p* = 0.037	*r* = 0.715 *p* = 0.004	*r* = 0.755 *p* = 0.002	-	-	*r* = 0.673 *p* = 0.033	-	-

Pearson's correlation coefficients (r) and p-values are showed.

CiTAS, Chemotherapy Induced Taste Alteration Scale; cSP1: contralateral early silent period; cSP2, contralateral late silent period; d, duration; EDX, electrodiagnostic examination; HRS, Hyposmia Rating Scale; iSP1, ipsilateral early silent period; iSP2, ipsilateral late silent period; l, latency; L, left stimulation; R, right stimulation; T0, baseline assessment; T1, 1-month follow-up; VAS-G, Gustatory Visual Analog Scale; VAS-O, Olfactory Visual Analog Scale.

### Clinical and electrophysiological follow-up at 20 months

The follow-up was performed in seven of 16 patients (Tables 1, 4, 5). In all but one patient (numbered as 13 in Tables 4, 5), the olfactory and gustatory deficits had totally regressed. The number of alterations at MIR examination was reduced in five patients (Table 4). Notably, in both patients with absent SP1 and SP2 at baseline, the inhibitory electrophysiological response was restored at follow-up. With regard to BR, R1 latency was bilaterally normalized in one of the two patients who presented prolonged values at baseline (Table 5). The amplitude of the R1 and R2 normalized at follow-up in all five patients who presented increased values either unilaterally or bilaterally at baseline.

## Discussion

In this study, we provide evidence of subclinical alterations of the trigeminal system in patients with COVID-19 and olfactory dysfunctions by means of the electrophysiological assessment of cranial nerve reflexes. The underlying abnormalities were more evident in the acute or subacute phases of the disease, although slight trigeminal impairment persisted in some subjects almost 2 years after symptom onset.

Masseter inhibitory reflex (MIR) is the most used neurophysiological tool for investigating the function of the third (mandibular) branch of the trigeminal nerve, which supplies sensory innervation of the buccal mucosa and inferior part of the face skin and contains motor fibers innervating the masticatory muscles (25). MIR consists of a reflex inhibition of the jaw-closing muscles elicited by a peri- or intra-oral electrical stimulation. Even after unilateral stimulation, MIR comprises an early and late component of suppression (SP1 and SP2, respectively) in both ipsilateral and contralateral masseter muscles. These silent periods are mediated by medium-myelinated Aβ afferents through oligosynaptic and polysynaptic circuits (for SP1 and SP2, respectively) within the brainstem (28, 29). On the other hand, the reflex arc of BR includes Aβ afferents of the first (ophthalmic) branch of the trigeminal nerve, which provides sensory innervation for the superior portion of the nasal cavity and the superior part of the face skin, as well as motor efferents of the facial nerve and brainstem neural circuits. BR comprises an early response (R1), which is ipsilateral to the side of stimulation and is relayed through a short oligosynaptic circuit, and a late response (R2), which is bilateral and is relayed through polysynaptic ponto-medullary pathways (25).

A relevant finding of this study is the prolonged latency of both the iSP1 and cSP1 after bilateral stimulation when assessing MIR in the patients' group, in the absence of significant differences in the latency of iSP2 and cSP2 as well as of early and late BR responses as compared with normal subjects. These results suggest a peripheral involvement of the mandibular branch rather than of the ophthalmic division of the trigeminal nerve. Indeed, in the case of a substantial brainstem impairment, we would have also expected greater latency alterations of MIR late components and additional BR abnormalities. Notwithstanding, limited brainstem damage cannot be ruled out at least in some patients, taking into account that assessment of the late components of both MIR and BR may be less sensitive in detecting subtle alterations. In fact, the SP2 at MIR investigation is often lacking even in physiological conditions, while the R2 components at BR examination are relatively unstable and they rapidly habituate to short-interval repetitive stimuli. Late responses are also less reliable considering that they are strongly modulated by suprasegmental afferents and can be influenced by cognitive factors (30, 31).

Our results deriving from BR evaluation differ from those recently described by Bocci et al. in patients with COVID-19 (32). These authors found marked alterations of the ipsilateral and contralateral R2 responses, which were absent or significantly reduced in amplitude and increased in duration, while no alterations were reported in the R1 response. Discrepancies with our findings of normal or only mildly impaired BR responses are not surprising considering the very different characteristics of the patients. Indeed, in the context of the severe disease course, all patients assessed in the study by Bocci et al. suffered from interstitial pneumonia and were intubated at the time of electrophysiological examination (32). Thus, the authors hypothesized a brainstem involvement featured by disruption of ponto-medullary circuitry subserving R2 responses and in close proximity to the respiratory nuclei.

The pathogenic mechanisms responsible for damage to the cranial nerves and brainstem structures in patients with COVID-19 remain controversial. To explain our findings, we hypothesize a peripheral involvement of the trigeminal nerve fibers due to either a direct viral attack or virus-induced inflammatory response. It is well-known that the angiotensin-converting enzyme 2 (ACE2) serves as the receptor for SARS-CoV-2 entry into host cells (30). Particularly, ACE2 is extensively expressed in the nasal and oral tissues (33, 34), thus damage to mucosal epithelial cells of the nasal and oral cavity may trigger a local inflammatory process capable to affect the trigeminal nerve endings. On the other hand, the trigeminal fibers could also be a potential direct target for SARS-CoV-2, as shown for other neurotropic respiratory viruses (35), and according to evidence of trigeminal nerve infestation by coronaviruses in mice (36). To define the underlying pathogenic mechanisms, without excluding the possible coexistence of direct and indirect damage to the trigeminal fibers, immunohistochemical staining of the trigeminal nerve branches in patients with COVID-19 could provide interesting information in future studies.

It has been supposed that SARS-CoV-2 could spread into the brainstem *via* the trigeminal nerve route (10). However, the prevailing hypothesis is that, in most subjects, neurological symptoms associated with COVID-19 do not arise from direct cytopathic effects mediated by SARS-CoV-2 but might result from a host-specific inflammatory response (9). Accordingly, in a recent systematic literature review, we showed that pathological correlates of neurological symptoms in patients with COVID-19 are mainly represented by brain inflammatory reactions and hypoxic-ischemic damage rather than neuronal viral load (8). Our electrophysiological findings do not support the hypothesis of brainstem damage, as we did not observe any clear evidence of alterations of the ponto-medullary polysynaptic circuits mediating the late responses of cranial reflexes. It is to remark, however, that the present results cannot be extended to patients with severe forms of the disease, in which brainstem damage related to SARS-CoV-2 neurotropism has been reported in some cases (37, 38).

The finding that more than 50% of the enrolled patients presented with headaches is in line with evidence that headache is a frequent symptom in patients with COVID-19 and smell disturbances (15, 39). To explain this link, neuroinflammation spreading from the olfactory bulbs to the frontal lobes has been supposed (37). Our data might also suggest that aberrant excitation of the trigeminal nerve fibers could contribute to headaches.

The increased amplitude of the R1 component at BR examination after unilateral stimulation, as well as other electrophysiological alterations observed only at single-subject analysis, such as the prolonged SP1 duration at MIR assessment found in several patients, could have different explanations. Indeed, beyond the possible influence of emotional and psychological factors including the arousal level (40–42), we cannot exclude the occurrence of subtle alterations in the ponto-medullary polysynaptic pathways or possible compensation mechanisms. In this regard, it is noteworthy that dynamic adaptation and compensatory processes in the interaction between the olfactory and trigeminal systems have been observed in patients with acquired anosmia, leading to an amplified trigeminal activation both at mucosal and central levels (43). These compensation mechanisms could develop over several days and this could explain the correlation between SP1 duration and time interval from symptom onset to electrodiagnostic examination. The correlation between SP1 duration and severity of smell and taste disturbances at 1-month follow-up also indicates that an increase in SP1 duration at baseline might predict more severe olfactory and gustatory deficits after 1 month. Though a long-term clinical and electrophysiological follow-up was performed only in a limited patient subgroup, our preliminary results suggest that the trigeminal alterations are not permanent but can recover over time in most of the subjects, even with *restitutio ad integrum*.

This study has some limitations. First, the number of patients enrolled is low, especially during the long-term follow-up. Furthermore, we did not investigate nasal and oral chemesthesis, which could have provided interesting clinical information on the trigeminal function to correlate with electrophysiological data. Among the strengths of this study, the recruitment of a patient's cohort with rather homogeneous clinical characteristics is noteworthy. However, the exclusion of patients with a severe disease course or without anosmia does not allow the data to be generalized to all patients with COVID-19.

In conclusion, this study is the first to prove subclinical impairment of the trigeminal nerve with an electrophysiological approach in patients with PCR-confirmed COVID-19 during the acute or subacute phases of the disease. Future electrophysiological studies on trigeminal function are needed to be carried out in larger patient groups having different clinical manifestations of disease, also considering the emergence of new SARS-CoV-2 variants that could have partly distinct pathogenic mechanisms. We believe that an in-depth understanding of the pathways involved in the neuronal damage associated with SARS-CoV-2 infection is essential for the development of effective prevention and treatment strategies.

## Data availability statement

The datasets presented in this study can be found in online repositories. The raw data used in this study are available in the Zenodo repository: https://zenodo.org/record/6414083#.

## Ethics statement

The studies involving human participants were reviewed and approved by Ethics Committee IRCCS Policlinico San Matteo. The patients/participants provided their written informed consent to participate in this study.

## Author contributions

GC: conceptualization (equal), investigation (equal), methodology (equal), supervision (equal), data curation (equal), formal analysis (lead), and writing–original draft preparation (lead). EM and MT: conceptualization (equal), investigation (equal), methodology (equal), data curation (equal), and writing–review and editing (equal). PP and EAn: investigation (equal), data curation (equal), and writing–review and editing (equal). GT and IQ: data curation (equal) and writing–review and editing (equal). SL, RD, AC, MB, AP, and CT: investigation (equal) and writing–review and editing (equal). EAl: conceptualization (equal), investigation (equal), methodology (equal), supervision (equal), data curation (equal), and writing–review and editing (equal). All authors contributed to the article and approved the submitted version.

## Funding

This study was supported by grants from the Italian Ministry of Health (Ricerca Finalizzata GR-2019-12369182).

## Conflict of interest

The authors declare that the research was conducted in the absence of any commercial or financial relationships that could be construed as a potential conflict of interest.

## Publisher's note

All claims expressed in this article are solely those of the authors and do not necessarily represent those of their affiliated organizations, or those of the publisher, the editors and the reviewers. Any product that may be evaluated in this article, or claim that may be made by its manufacturer, is not guaranteed or endorsed by the publisher.
